# Carbon Fiber—Silica Aerogel Composite with Enhanced Structural and Mechanical Properties Based on Water Glass and Ambient Pressure Drying

**DOI:** 10.3390/nano11020258

**Published:** 2021-01-20

**Authors:** Agnieszka Ślosarczyk

**Affiliations:** Institute of Building Engineering, Faculty of Civil and Transport Engineering, Poznan University of Technology, Piotrowo 3, 60-965 Poznań, Poland; agnieszka.slosarczyk@put.poznan.pl; Tel.: +48-61-665-2166

**Keywords:** silica aerogel, water glass, ambient pressure drying APD, carbon microfibers: structural and mechanical properties

## Abstract

The article presents the synthesis of silica aerogel from a much cheaper precursor of water glass that was reinforced with short pitch carbon fiber by way of ambient pressure drying. Before being added to the silica gel, the carbon fibers were surface modified to increase adhesion at the interfacial border. We were able to obtain stable structures of the composite with the amount of fibers above 10% by volume. The presence of fibers in the silica matrix resulted in lower synthesis time of the composite, improved adhesion of fibers to the aerogel nanostructure, and increased mechanical and structural parameters. An additional effect of the presence of fibers in excess of 10% by volume was a new function of the nanocomposite—the ability to conduct electric current. The most optimal parameters of the composite, however, were obtained for silica aerogel reinforced with 10 vol.% of carbon fibers. This material indicated relatively low density and good physical parameters. The paper also analyzes the results on the synthesis of fiber-reinforced silica aerogels that have appeared in recent years and compares these to the results gained in presented work.

## 1. Introduction

**Figure 1:**
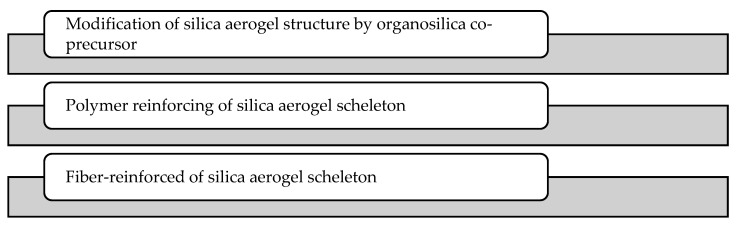
Most frequently used ways of reinforcing the structure of silica aerogels.

The widespread interest in the synthesis of silica aerogels and their use in many applications, including the thermal and sound insulators [1,2], catalysts and carriers of various active substances [3,4,5], adsorbents and absorbents [6,7,8,9] is due to their unique lightweight nanostructure and properties such as low thermal and sound conduction coefficient, high porosity, and low light reflectance [3,10,11]. Nevertheless, despite many positive features, silica aerogels are characterized by low fracture toughness, which very often determines their wider use in many industries. The solution to this problem can be an appropriate modification of particular stages of silica aerogels preparation (synthesis, ageing, drying) and selection of a suitable method of its structure reinforcement leading to the composite characterized by satisfactory chemical and physical parameters. The most frequently used ways of reinforcing the structure of silica aerogels are presented in Figure 1.

Structural and mechanical features of the gel can be modified in ageing stage by dissolving and repeatedly precipitating the silica from the surface of particles on to the borderline particle—particle and connecting and/or precipitating oligomers, which were unreacted during gelling. Another method assumes adding extra amount of precursor and co-precursor to the solution before and after the moment of gelation, so that it builds into the structure of the gel and, thus, reinforces it [12,13,14].

Apart from altering parameters of the synthesis, the mechanical properties of silica aerogels can be modified by implementing various additives to their structure, e.g., nanoparticles and metal nano-oxides or by applying reinforcement in the form of a short structural fiber or fiber mats [15,16,17,18].

There is also research carried out on covering the surface of silica aerogels with polymers [19,20,21]. This action is taken before the stage of drying the gel; as a result, the surface of silica aerogel is covered with a layer of polymer that increases the resistance of silica structure to breaking. Nevertheless, the easiest way of increasing the strength of silica aerogel is applying fiber reinforcement processes. The main purpose of using short fibers in dispersed or long mat form is to strengthen the structure of the silica aerogel. Silica aerogels are characterized by good structural parameters and relatively good compressive strength, but they break very easily. This is characteristic of all the so-called brittle matrices that break when the maximum stress is reached. The role of the fibers is to strengthen the structure of the aerogel by bridging the cracks that arise during increasing stress, and, depending on the parameters of the fibers, transferring the load through the fibers.

The properties of silica aerogel/fiber composites depend on the type of fibers and the interaction between the fiber and matrix. Important parameters are: fiber size and shape factors, share and distribution of fibers in the aerogel matrix, and interaction at the fiber/silica matrix interface. Exemplary physical and mechanical parameters of composites depending on the type of fiber are presented in Table 1 [22,23,24,25,26,27,28,29,30,31,32,33,34]. So far, ceramic fibers, nanotubes, and nanotubes have been used most often for strengthening the aerogel structure, as ceramic fibers and nanostructures significantly improved the compressive strength of the composite, which range from 0.030, to 0.483 MPa—depending on the preparation way and the type of fiber. In the case of long ceramic fibers, a very high compressive strength is obtained in comparison with other fibers and nanofibers (over 6 MPa). On the other hand, ceramic fibers significantly worsen the density and other structural parameters of composites. Positive effects on the mechanical parameters of aerogel composites were also observed with the use of aramid and nanofiber polymers, e.g., of polyvinyl (PVA), for which the maximum values of compressive strength were 0.043 MPa and 5.23 MPa, respectively. For aerogels reinforced with polyaniline fibers, tensile strengths ranging from 0.04, to 0.06 MPa were tested. Cellulose nanofibers and carbon nanotubes were also used to reinforce the structure of the aerogel, obtaining an improvement in mechanical properties, compressive strength and maximum load, which for these composites were 2 MPa and 300 N, respectively. An interesting solution is the combination of glass and carbon fibers in a sandwich structure. Carbon fibers ranging from 5 to 15% were used as a layer between two layers of fiber glass. Studies have shown the positive effect of such an arrangement on both mechanical properties and optimal structural-insulation parameters. Tensile strengths ranged from 2.889–4.39 MPa, while thermal conductivity varied from 0.031 to 0.052 depending on the amount of fibers. Recent studies indicate that organic fibers are increasingly used to reinforce silica aerogel. The advantage of these fibers is low density, small diameters, and much greater flexibility. The studies also indicate better compatibility of polymeric fibers with the aerogel matrix, which may lead into improved structural parameters and less dusting of the composite [35]. This group also includes carbon fibers, which are characterized by very good chemical and physical parameters, particularly with regard to density. Unlike most polymeric fibers, they are resistant to increased temperatures and can be surface modified.

Therefore, in the presented article, cheap carbon fibers obtained from carbon tar pitch were used to strengthen the nanostructure of silica aerogel. Prior to their use in silica aerogel matrix, carbon fibers were surface modified in a nitric acid solution to increase the amount of hydrophilic functional groups on their surface. The presented article is a consequence of previously initiated research describing the nanocomposites based on short carbon fiber and silica aerogels based on water glass solution [36]. In contrast to previous studies, in this paper, the temperature of silica aerogels synthesis were decreased and different amounts of carbon fibers determining the form of the composite were applied. The paper focuses mainly on the structural properties of the obtained materials and their correlation with the mechanical and insulating parameters. The paper also contains an in-depth analysis of mechanical and structural parameters obtained for fiber-reinforced silica aerogels that have been published by other research centers recently. These were compared with the results obtained in this paper.

## 2. Materials and Methods

The synthesis of silica aerogel–carbon fiber composite was carried out by sol-gel method using a 10% aqueous water glass solution in the presence of 1 mol/dm^3^ acid catalyst (citric acid). Carbon fibers with a diameter of 13 µm and a length of 700 µm (OSAKA GAS Co., Osaka, Japan) in an amount of 1 to 15% by volume were added to the aqueous glass solution before the gelation moment. In order to obtain a good dispersion of the fibers in the sol, the carbon fibers were modified in concentrated nitric acid at 105 °C. Oxidation of the fiber surface was carried out for 5 h. After treatment, the fiber specific surface area changed from 2 to 10 m^2^/g. The aging step was conducted for 24 h in methanol aqueous solution followed by 7 days in methanol. The resulting composites were then dried in a 1:4 mixture of TMCS/n-hexane for 24 h followed by air drying.

Porous structure parameters of silica aerogel composites (specific surface area, pore diameter, total pore volume) were determined on the basis of adsorption-desorption isotherms of nitrogen vapors using ASAP 2020 analyzer (Micromeritics Co., Livermore, CA, USA), while temperature stability was determined using the Jupiter STA 449F3 apparatus (Netzsch GmbH, Selb, Germany) in the temperature range 30–1000 °C, under a nitrogen atmosphere. Bulk density of the composites was determined as a mass to volume ratio on cylindrical samples.

Mechanical characterization of the manufactured composites was carried out using an Instron electromechanical press, ElectroPulsTM E10000, class 0.5. The test was performed on 5 samples of silica aerogel–carbon fibers composite with 10 and 15 vol.% of fibers. The microstructure of silica aerogel–carbon fiber composites was studied by Tescan–3–Vega microscope (Tescan, Czech Republic). Specimens were sputter coated with Au/Pd nanolayer prior to testing.

The thermal conduction coefficient for composites silica aerogel–carbon fibers was made using the stationary method, by means of the FOX50 TA Instruments. Measurement temperature was 10 °C. The conductivity of silica aerogel composites was determined by means of potentiostat/galvanostat VMP3 (Bio-Logic, Seyssinet-Pariset, France). Measurements were taken in a Swagelok^®^ cell using an electrochemical impedance spectroscopy (EIS) and linear sweep voltammetry (LSV) techniques.

## 3. Results

On the basis of literature study and preliminary research, it was assumed that the sol-gel method with the use of organosilicon compounds of tetramethyl orthosilicate type (TMOS) and tetraethyl orthosilicate type (TEOS) will contribute to producing silica aerogel–carbon fiber composites with the assumed physicochemical parameters. Drying in supercritical and atmospheric conditions was applied and comparable physicochemical parameters of nanocomposites were obtained [37,38]. Based on the positive results of the research, a decision was made that in the main phase of the research, water glass, which is far cheaper than organosilicon compounds, was to be used as the precursor of the silica aerogel, and the drying was to be conducted only under atmospheric conditions. This eliminates the more costly supercritical drying that limits aerogel production on a technical scale. Due to the potential use of silica aerogels in the construction industry, citric acid was used as a catalyst for the sol-gel reaction instead of the most commonly used hydrochloric acid. The literature study indicated that there are better physical parameters of silica aerogels created in the presence of weak organic acids than those generated in the presence of strong inorganic acids, e.g., hydrochloric acid, whose contiguity may negatively affect other construction materials, like concrete or steel [39]. The purpose of this phase of research was to determine conditions and methods of synthesis of silica aerogel. Eventually, the decision was made to conduct a much faster one-step synthesis from water glass solution, excluding the preliminary phase, i.e., obtaining silicic acid, which may allow for an easier and cheaper method of future synthesis of this nanocomposite.

**Figure 2:**
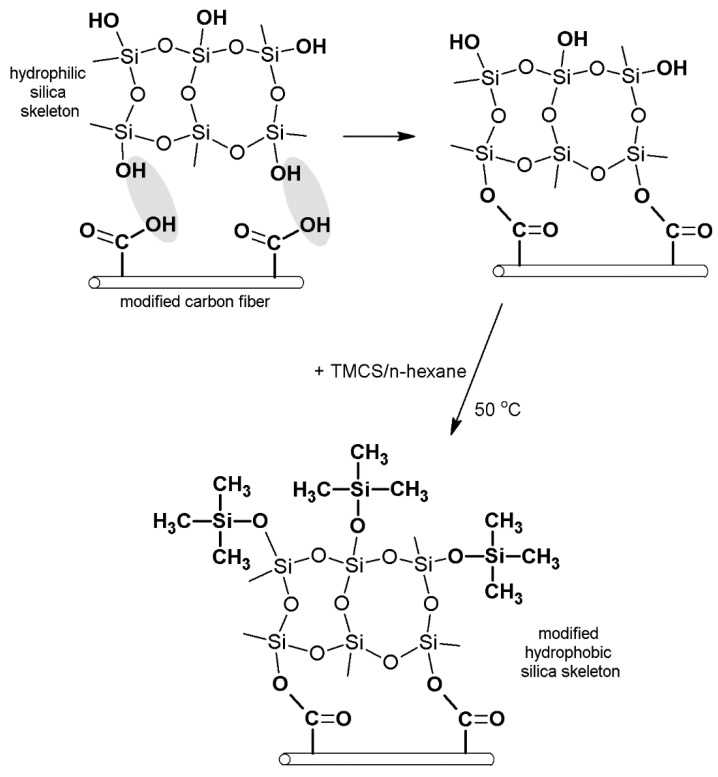
Synthesis of the silica aerogel–carbon fibers composite.

Drying in atmospheric conditions was, hence, applied; this included surface modification of silica aerogel with trimethylsilyl chloride (TMCS) and employment of carbon fibers to reinforce the structure of aerogel. The used coal fibers came from the much cheaper carbon precursor, coal tar pitch, which is characterized by a relatively high tensile modulus to bulk density, good heat stability up to 600 °C, compatibility, and good electrical conductivity. Carbon fibers were the subject of surface modification in nitric acid to increase the share of oxygen functional groups on their surface and to enhance the number of connections between the silica structure and carbon fibers. The scheme of the composite synthesis is presented in Figure 2. It was assumed that simultaneous modification of silica aerogel with trimethylsilyl chloride and carbon fibers will help obtain the desired structural and mechanical parameters of the composite and quicken the process of synthesis of the nanomaterial. Moreover, we thought that the reaction between carbon fibers and silica structure should result in lowering the contraction of silica aerogel while drying and limit the dustiness of the composite, which occurs in aerogel mats currently produced with the use of glass and polymer fibers. Implementing carbon fibers into the structure of silica aerogel will also, we concluded, result in obtaining new functions of the material, e.g., its ability to current conductivity.

### 3.1. Structural and Chemical Characterization of Silica Aerogel

The analysis of the results presented in the Table 2 shows that a smaller amounts of fibers, 1 and 5 vol.% shares, increases the density of the composite and the specific surface area, whereas the bigger amounts, 10 and 15 vol.%, result in decreasing the density of the material, giving lower values of the specific surface area than the pure silica aerogel. The presence of carbon fibers in the structure of the aerogel positively affects shrinkage during drying, the greater number of fibers induces a decrease of volume shrinkage of the material. The best structural parameters were obtained for aerogel with a 5 vol.% addition of carbon fibers, however, the sample had extensive microcracking. Only when the addition of fibers reached 10 vol.% did the composite have dense structure without microcracking.

**Figure 3:**
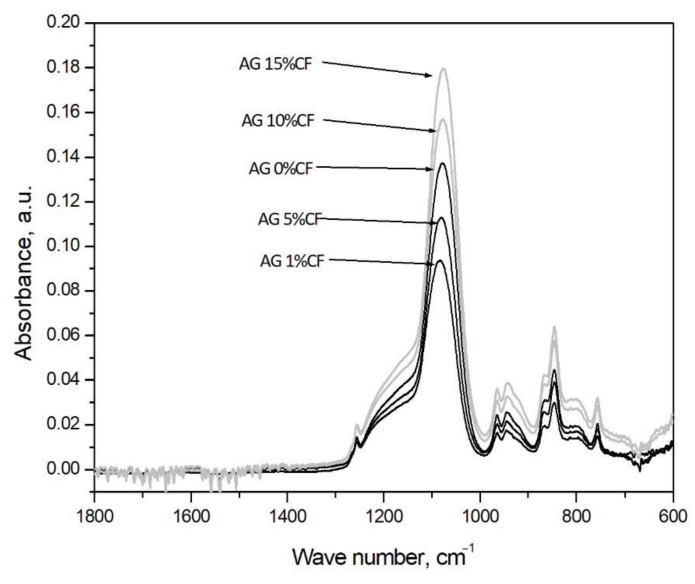
FTIR analysis curves obtained for silica aerogel and its carbon fiber composites in relation to the amount of fibers (precursor 10% solution of water glass, APD).

Figure 3 presents the infrared spectra registered for silica aerogels and silica aerogel reinforced with carbon fiber in relation to the number of fibers. In case of all materials, according to preliminary research and results for TEOS and TMOS with wave number around 1100 cm^−1^, there is a band appearing with Si-O-Si bonds of the silica structure [40]. In the case of fiber composites in the amount of 1 and 5 vol.%, the band intensity decreases; for fiber composites in the amount of 10–15 vol.%, the band intensity increases. The greater number of Si-O-Si bond in composites with 10–15 vol.% of fibers may indirectly indicate reaction between the silica structure and the surface area of carbon fibers from which the increase of the silica chain begins. Furthermore, in all cases, there were bands at wave numbers 1258 cm^−1^, 847 cm^−1^, and 758 cm^−1^ that can be assigned to Si-C bonding, and that is connected with TMCS modification of the gel [41,42].

**Figure 4:**
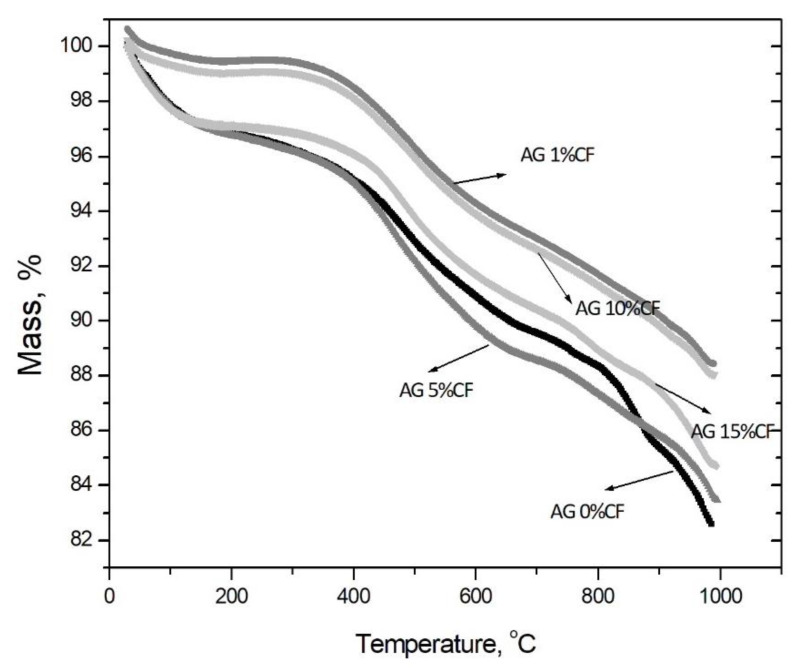
Thermogravimetric curves obtained for silica aerogel and its composites with carbon fibers in relations to the amount of fibers (precursor 10% solution of water glass, APD).

The thermogravimetric analysis presented in Figure 4 shows that higher heat stability is characteristic for composites with 1 and 10 vol.% addition of carbon fibers, in that there was a 2% loss of weight at the temperature up to 400 °C, whereas other configurations had a 4% loss. Nevertheless, all materials showed high heat stability up to 700 °C. The physicochemical analysis of silica aerogel/carbon fiber composites showed that durable structures with high heat stability were obtained with carbon fiber contents of 10 and 15 vol.%. These showed a 6 and 10% loss of weight, respectively, and proved the effective modification of the structure in TMCS. The explanation for the unusual behavior of composites with the addition of different amounts of fibers is the distribution of fibers in the aerogel matrix. These results are consistent with the structural analysis. When 1 vol.% fibers were added to the silica aerogel, the fibers were homogeneously dispersed in the matrix. Due to the introduction of a small amount of fibers, the specific surface area of the composite increased, this trend was also maintained for 5 vol.% carbon fiber addition, but at this ratio the fibers did not disperse uniformly. There were places where some fibers agglomerated and there was no good adhesion between the fiber surface and the silica matrix. It should be noted here that both structures were unstable and fractured and disintegrated into granular form. Only 10 vol.% addition of carbon fibers made it possible to obtain a stable aerogel structure; nevertheless, at this amount of fibers a decrease in the specific surface area of the composite was observed. This is due to the much lower value of the carbon fiber specific surface area of about 10 m^2^/g. A further decrease in the specific surface area of the aerogel composite was observed when fibers were added at 15 vol.%. Despite the durable structure, this amount of fibers was already large enough to significantly deteriorate the temperature stability of the composite. As with the 5 vol.% addition in this configuration, the carbon fibers tended to agglomerate and interfere with the formation of a homogeneous silica aerogel structure. The similar tendencies were observed by Horg et al. [34].

### 3.2. Mechanical and Microstructural Characterization of Silica Aerogel Composite

**Figure 5:**
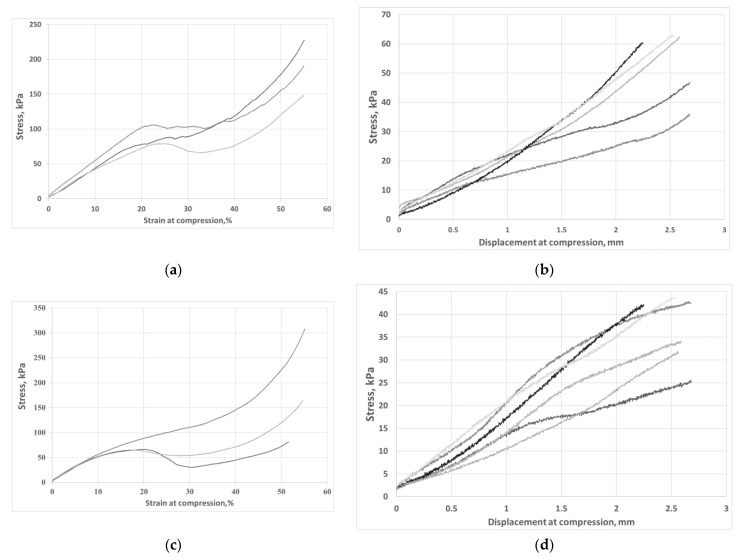
Stress-strain and stress-displacement curves obtained for AG 10% CF (**a**,**b**) and for AG 15% CF (**c**,**d**), respectively.

The purpose of using carbon fibers was to strengthen the structure of the silica aerogel. Physicochemical analysis showed that 10 and 15 percent addition of fibers brought about a permanent composite structure without evident cracking. In the case of smaller amounts of fibers, the material structure was very brittle and unsuitable for mechanical testing. Therefore, mechanical analysis of the composites was carried out for two materials with the highest content of fibers marked as AG 10% CF and AG 15% CF. In the first stage, the composite samples were loaded to the maximum deformation in uniaxial compression at the rate of stress increase of 1 mm/min. On the basis of stress-strain curves, the elastic range of the material was determined, and, in the second stage, the dependence of stress on deformation and stress on displacement in the elastic range was investigated. From the stress-deformation curves, the maximum values of compression strength were obtained. Additionally, on the basis of the height of the samples before and after the compressive load in the elastic range, the return elasticity of the composites was assessed. This is a physical characteristic that defines the ability of a material to recover its original shape and volume after the removal of external deformation forces. The stress-strain curves in compression are shown in Figure 5a,c, respectively, for aerogel composites with 10 and 15 percent added carbon fibers. For both composites, three ranges are visible on the curves: the first range—elastic, determining the linear increase in stress up to 20% of the specimen strain, the second range—extra-elastic—from 20 to 30% of the specimen strain (plastic-elastic), and the third range above 30% of the strain, during which the aerogel structure compacts and gradual material degradation occurs. Similar material characteristics were obtained in other composites based on silica aerogel and fibers, which were described in detail by Yang, Li, Sedova, and others [see Table 1].

**Figure 6:**
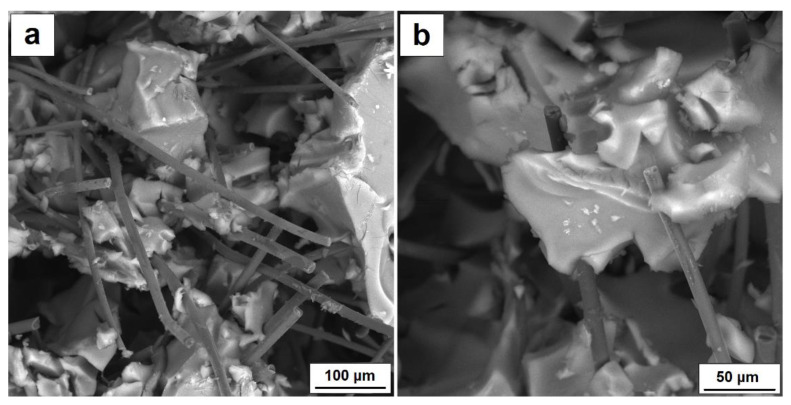
(**a**) SEM characterization for AG 10% CF composite, (**b**) bridging of silica aerogel by carbon fiber.

On analyzing the stress-strain curves for composites with 10 and 15 percent fiber addition, it can be seen that a composite with lower content shows higher stress values in the range up to 20 percent of the specimen strain than does a composite with more fibers. Nevertheless, in order to compare the mechanical characteristics of both composites, the strain range up to 20% was adopted for further studies and the dependence of stress on displacement was determined (Figure 5b,d). The values of compressive strength and return elasticity are shown in Table 3. The aerogel composite with 10% fiber addition had much higher compressive strength, even though both materials showed similar deformations, and when the load was removed, the specimens returned to their original shapes at 93%. For both materials, the specimen deflection at strain range up to 20% was 2.5 mm. Better parameters of the composite with the addition of 10% vol. carbon fibers are the result of the homogeneous structure of the composite; in this case, higher structural parameters of the aerogel and higher porosity were obtained. Most of the carbon fibers act as a strengthening of the aerogel structure by bridging the resulting micro-rises, as shown in Figure 6a,b. The presence of higher fiber shares in the aerogel matrix means that not all the fibers are bound to the silica skeleton and form micropores in the structure that weaken the structure of the composite mechanically. Strength and structural results clearly indicate that more favorable distribution of carbon fibers in the aerogel matrix was obtained for AG 10% CF composite.

Table 3 also lists the values of electrical conductivity of the silica aerogel reinforced with carbon fibers in volumes of 10 and 15%. In order to define the conductivity of the tested materials, Electrochemical Impedance Spectroscopy was used. Nonconductive materials can conduct current as a result of introducing conductive fillers such as particles or fibers into their structure in such numbers that the so-called percolation threshold is reached. This is the amount of fillers at which particles or fibers come into contact with each other to form a specific conductive network in a nonconductive material. It is easier to obtain the percolation threshold for fibers than it is for spherical particles—this is possible even by way of a few percent addition and depends strongly on the structural parameters of the fibers and on the properties of the matrix. It is much more challenging to achieve the percolation threshold for dielectric materials, such as silica aerogels. In this case, the percolation threshold allowing creation of fiber net was obtained by a 10 percent addition of fibers. Figure 6 presents the microstructure of the aerogel composite with 10% fiber addition.

While the photo shows fiber connections, part of the fibers separated from each other by the aerogel structure remains inactive. The 15% fiber addition of fibers definitely ensures good electrical conductivity—in this case, it was three times higher in comparison with 10% fiber composite, and equaled 0.055 mS/cm. Both the aerogel matrix and smaller amounts of fibers of 1 and 5% volume did not conduct current. Similar values of conductivity were gained for aerogel composites with the addition of conductive polymer—polyaniline [23]. The highest conductivity of 0.022 mS/cm was obtained for 12 mg/mL of polyaniline, which equaled the amount of 16.5 wt.%.

Lightweight silica aerogel composites—reinforced with short fiber or nanofiber, due to their very large specific surface area and low volumetric density, which translates into a low heat conduction coefficient—are ideal for both low and high temperature insulation. The main factors influencing the change in the conductivity of a silica aerogel are the type of fiber carrier used, mainly the fiber density, specific surface area, and conductivity of the fibers themselves. The results of the heat conduction coefficient obtained in the study for the composite silica aerogel based on water glass, reinforced with carbon fibers in the amount of 10 and 15% vol, are listed in Table 4 [43]. Additionally, the table shows the results of heat conductivity coefficients for composites of silica aerogel reinforced with other types of fibers and nanofibers for comparison purposes [25,26,27,29,30,31,32,34]. Special attention was paid to the correlation of heat conduction coefficient values with density and specific surface area of nanocomposites. The values of heat conduction coefficient for the tested composites AG 10% CF and AG 15% CF depended on the number of fibers and structural properties of the aerogel, being 0.0325 and 0.332 W/(m × K), respectively. A higher proportion of fibers was found to prevent the formation of a homogeneous aerogel structure, which results in a decrease in the specific surface area of the composite and lower heat conduction coefficient values. Similar trends were observed by other researchers—as the share of both fibers and nanofibers increased, the physicochemical properties of aerogels deteriorated and the values of heat conduction coefficients decreased, regardless of the method of synthesis and silicon precursor. Nevertheless, the obtained values of the thermal conductivity coefficient were similar to those obtained by other researchers (Table 4).

### 3.3. SEM Characterization of Silica Aerogel Composite

**Figure 7:**
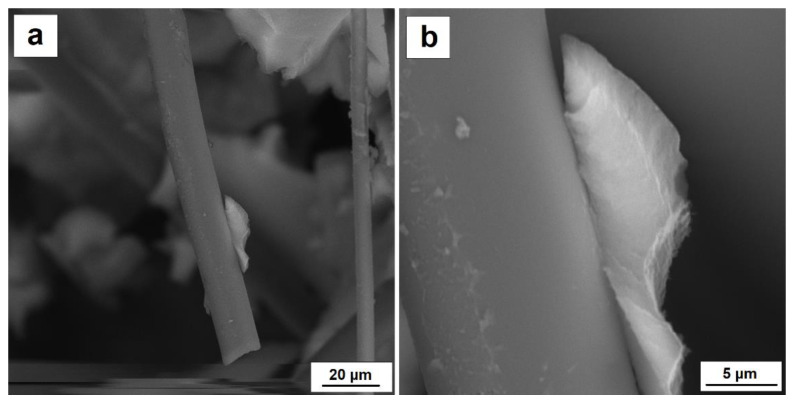
(**a**) Adhesion of silica aerogel to carbon fiber, (**b**) boundary between silica aerogel and carbon fiber.

**Figure 8:**
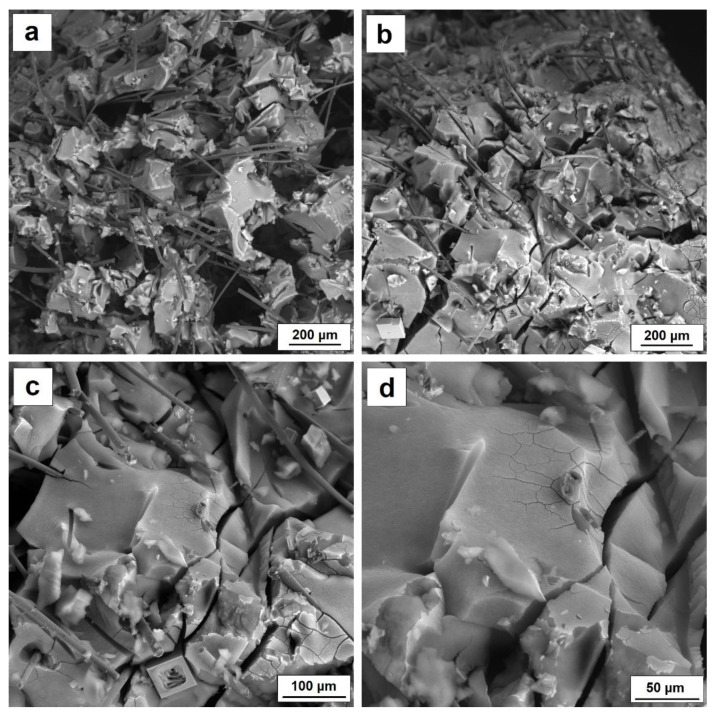
(**a**) SEM characterization of AG 10% CF before loading, (**b**) and after loading, (**c**) microcracking of silica aerogel structure reinforced with carbon fiber, (**d**) enlargement of silica aerogel microcracks.

Selected images of the microstructure of the composite silica aerogel–carbon fibers in a share of more than 10% by volume and the adhesion on the boundary between silica aerogel and carbon fibers can be found in Figure 7 and Figure 8. The SEM micrographs presented in Figure 7 show a single carbon fiber and the phase boundary between carbon fiber and silica aerogel. The SEM image indicates very good adhesion of the aerogel to the fiber surface, which is the result of the reaction between the oxidized fiber surface and the hydroxide groups located on the silica gel surface.

While, the SEM photo presented in the Figure 8a shows two interpenetrating structures of silica aerogel and carbon fiber. In addition, pores with diameters between 50 and 100 µm are visible in the structure, probably due to gel contraction during drying and to the limitation of the formation of a homogeneous aerogel structure by the presence of large shares of carbon fibers. Figure 8 b also reveals the microstructure of the composite silica aerogel–carbon fiber after loading. In comparison with Figure 8a, which shows the microstructure of the composite before loading, the picture show a significant densification of the structure and directional arrangement of the fibers. Additionally, microcracking in the structure of the aerogel itself is visible (Figure 8c,d). SEM images confirm the strength results and return elasticity values. Silica aerogel–carbon fiber composite does not show a linear relationship between stress and deformation in the elastic range. After exceeding 10% deformation, the composite is compacted and the structure is permanently micro-cracked, which means that the material does not return to its original shapes and dimensions after load removal.

## 4. Conclusions

Wide physicochemical analysis provides evidence that it was possible to gain silica aerogel–carbon fiber composites in a simple, one-stage process with the use of atmospheric drying. Best structural and insulating parameters were obtained for silica aerogel with 10% addition of 0.199 g/cm^3^, specific surface 474.3 m^2^/g, and thermal conductivity coefficient around 0.0325 W/m·K. Moreover, the material presented high resistance to temperature, having stable structure up to 400 °C. This outcome is due to the effect of simultaneous modification of the silica gel structure with carbon fibers and surface modification in the mixture of TMCS/n-hexane in 50 °C. Hydrophilic functional groups located on the surface of the oxidized fibers, after the silicon precursor was introduced, are additional active centers where the gelation process and creation of silica gel chains begin. Thus, we receive two interfluent nets—a silica net and a net created by carbon fibers making homogeneous mesoporous structure.

Application of greater amount of fibers—above 10 vol.%—enabled shortening the process of modification of silica gel in the mixture of TMCS/n-hexane down to 24 h and contributed to receiving a stable structure of the nanocomposite with lowered contraction during drying and good mechanical parameters.

Short carbon fibers from a cheaper precursor—coal tar pitch—were applied as the reinforcement for silica aerogel for the first time. Fibers applied in this work combine the advantages of inorganic fibers—they also have equally high temperature stability, and polymer fibers—they have beneficial strength parameters and relatively low bulk density. Moreover, carbon fibers are biocompatible and as a result of surface modification, they can achieve solid connections with silica skeleton, which results in better parameters of the final composite and limits dusting. Additionally, as a result of introduction of carbon fibers into the silica aerogel structure, along reinforcement of the structure, a new feature of the material was gained—electrical conductivity. Percolation threshold was gained for 10 vol.% of fibers; for this amount, the fibers created a conductive net within the dielectric material—producing electric conductivity equal to 0.015 mS/cm. Greater amount of fibers (15 vol.%) induced over three times higher electric conductivity.

The research outcome indicates that insulating and mechanical properties of the received composite of silica aerogel and carbon fibers mainly depend on the structural properties of the silica aerogel, especially its porosity. Carbon fibers, depending on their volume, can either strengthen such features or weaken them. Via proper modification of the silica aerogel microstructure and the fiber content, it will be possible in the future to shape specific material properties, depending on potential application. The research results also prove the significant cognitive meaning, the originality of the subject, and possibility of practical application of the received materials.

## Figures and Tables

**Table 1:** Physical and chemical characteristic of silica aerogel–carbon fibers composites in relations to the amount of fibers.

Precursor/ Conditions of Synthesis	Bulk Density, g/cm^3^	Fiber Type/Amount of Fibers	Flexural f_f_ or Compressive Strength f_c_, MPa	Reference
TEOS/APD/TMCS	0.202	Nanofibers PVA	f_f_ = 1.1 f_c_ = 5.23	[22]
TMOS/LTSD/CO_2_	0.071–0.079	Polyaniline nanofibers	f_f_ = 0.04–0.06	[23]
Water glass/APD/TMCS	0.23	Multi-walled carbon nanotubes	Max load 300 N	[24]
TEOS/APD/TMCS	0.154–1.193	Silica nanofibers	f_c_ = 3.5	[25]
Water glass/APD/TMCS	0.104–0.146	Silica nanofibers	f_c_ = 0.16	[26]
TEOS/APD/TMCS	0.15–0.17	Aramid fibers	f_c_ = 0.019–0.043	[27]
TEOS/LTSD/CO_2_	No data	WS_2_ nanofibers	f_c_ = 0.03	[28]
TEOS/APD	0.18–0.12	Ceramic nanotubes	f_c_ = 0.4–1.45	[29]
TEOS, ZrOCl2/HTSD/ethanol	0.274–0.419	Ceramic fibers	f_c_ = 0.286–0.438	[30]
TEOS/APD/TMCS	0.1–0.3	Cellulosic nanofibers	f_c_ = 0.17–2	[31]
TEOS/APD/TMCS	0.15–0.162	Aramid fibers	f_c_ = 0.83–1.22	[32]
TEOS/HTSD/ethanol	0.29	Ceramic fibers	f_c_ = 6.3	[33]
TEOS/APD/TMCS	0.131–0.245	Glass/carbon fibers	f_f_ = 2.889–4.39	[34]

**Table 2:** Physical and chemical characteristic of silica aerogel–carbon fibers composites in relations to the amount of fibers.

Sample	Density, g/cm^3^	Volume Shrinkage %	Specific Surface Area, m^2^/g	Average Pore Diameter, nm	Average Pore Volume, cm^3^/g
AG0%CF	0.209	granulate	496.5	10.2	1.271
AG1%CF	0.336	67.5 + extensive microcracking	551.0	12.2	1.694
AG5%CF	0.233	52.3 + microcracking	571.3	14.6	2.091
AG10%CF	0.199	44.6	474.6	14.5	1.724
AG15%CF	0.225	44.4	467.0	12.7	1.486

**Table 3:** Electrical conductivity and mechanical characteristic of silica aerogel–carbon fibers composites in relations to the amount of fibers.

Parameter	AG10%CF	AG15%CF
Compressive strength, MPa	0.054 ± 0.012	0.038 ± 0.008
Resilience, %	93.3	93.3
Electrical conductivity, mS/cm	0.015 ± 0.005	0.055 ± 0.005

**Table 4:** Thermal conductivity coefficient of selected silica aerogel–carbon fibers composites in relation to synthesis parameters and densities.

Precursor/ Synthesis Conditions/Type of Fiber	Volume Density g/cm^3^/Specific Surface Area m^2^/g	Thermal Conductivity Coefficient, W/(m·K)	Literature
Water glass/APD/TMCS/carbon fibers 10 vol.%	0.199/474.6	0.0325	This work, 43
Water glass/APD/TMCS/carbon fibers 15 vol.%	0.225/467.0	0.0332	This work, 43
TEOS/APD/TMCS/silica nanonfibers	0.154–0.193/851–899.5	0.022–0.027	25
TEOS/APD/TMCS/aramid fibers	0.14–0.17	0.022	27
TEOS/APD/ceramic nanotubes	0.12–0.18/346–526	0.028–0.038	29
TEOS, ZrOCl_2_/HTSD ethanol/ceramic nanotubes	0.274–0.419	0.0277–0.0273	30
TEOS/APD/TMCS/cellulose fibers	0.1	0.0226	31
TEOS/APD/TMCS/aramid fibers	0.150–0.162/955	0.023–0.028	32
Water glass/APD/TMCS/silica nanofibers	0.104–0.146/799–626	0.021–0.023	26
TEOS/APD/TMCS/glass/carbon fibers	0.131–0.245/933–335.3	0.031–0.0513	34

## Data Availability

The data presented in this study are available on request from the corresponding author.
